# PhaP phasins play a principal role in poly-β-hydroxybutyrate accumulation in free-living *Bradyrhizobium japonicum*

**DOI:** 10.1186/1471-2180-13-290

**Published:** 2013-12-11

**Authors:** Ken-ichi Yoshida, Yuki Takemoto, Takayuki Sotsuka, Kosei Tanaka, Shinji Takenaka

**Affiliations:** 1 Department of Agrobioscience, Graduate School of Agricultural Science, Kobe University, 1-1 Rokkodai, Nada, Kobe 657 8501, Japan; 2 Organization of Advanced Science and Technology, Kobe University, 1-1 Rokkodai, Nada, Kobe 657 8501, Japan

**Keywords:** *Bradyrhizobium japonicum*, Phasin, PHB

## Abstract

**Background:**

*Bradyrhizobium japonicum* USDA110, a soybean symbiont, is capable of accumulating a large amount of poly-β-hydroxybutyrate (PHB) as an intracellular carbon storage polymer during free-living growth. Within the genome of USDA110, there are a number of genes annotated as paralogs of proteins involved in PHB metabolism, including its biosynthesis, degradation, and stabilization of its granules. They include two *phbA* paralogs encoding 3-ketoacyl-CoA thiolase, two *phbB* paralogs encoding acetoacetylCoA reductase, five *phbC* paralogs encoding PHB synthase, two *phaZ* paralogs encoding PHB depolymerase, at least four *phaP* phasin paralogs for stabilization of PHB granules, and one *phaR* encoding a putative transcriptional repressor to control *phaP* expression.

**Results:**

Quantitative reverse-transcriptase PCR analyses of RNA samples prepared from cells grown using three different media revealed that PHB accumulation was related neither to redundancy nor expression levels of the *phbA*, *phbB*, *phbC*, and *phaZ* paralogs for PHB-synthesis and degradation. On the other hand, at least three of the *phaP* paralogs, involved in the growth and stabilization of PHB granules, were induced under PHB accumulating conditions. Moreover, the most prominently induced phasin exhibited the highest affinity to PHB in vitro; it was able to displace PhaR previously bound to PHB.

**Conclusions:**

These results suggest that PHB accumulation in free-living *B. japonicum* USDA110 may not be achieved by controlling production and degradation of PHB. In contrast, it is achieved by stabilizing granules autonomously produced in an environment of excess carbon sources together with restricted nitrogen sources.

## Background

Poly-β-hydroxybutyrate (PHB) is a polymer used for the storage of carbon and energy in a large variety of prokaryotes. It is accumulated in the cytoplasm if a carbon source is provided in excess and if any other essential nutrient is limited [1]. PHB belongs to the polyesters class of polymers, which is of interest as an industrial plastic because of its biodegradability and origin from renewable resources. Microbial PHB synthesis is a promising strategy for the production of bioplastics and offers a promising opportunity to transition toward a future-oriented bioeconomy [2].

Most species of rhizobia synthesize PHB and accumulate it in intracellular granules [3]. In some species, PHB accumulation can exceed 50% of the cell’s dry weight [4,5]. Various ways that rhizobia can use PHB to benefit their plant hosts have been proposed. For instance, it was proposed that PHB utilization could sustain the oxygen demand of the bacteroids during darkness; thus, contributing to the preservation of nodule activity and the continuation of nitrogen fixation at high rates [6]. PHB may also fuel the differentiation of rhizobia into nitrogen-fixing bacteroids [7]. In addition, rhizobia may simply degrade PHB in ways that enhance their own fitness. PHB may provide the energy and carbon required for bacterial reproduction, or for stress tolerance required within senescing nodules or after symbiotic rhizobia escape into the soil and transition to the free-living state.

Biochemically, PHB synthesis can compete with nitrogen fixation [1]. In addition, a negative correlation was observed between the rate of nitrogen fixation and PHB accumulation [8,9]. A mutant of *Rhizobium etli*, that did not accumulate PHB, was shown to significantly fix more nitrogen than the isogenic wild type [10,11], whereas non-fixing *nifH* mutants of *R. etli*[12] and *Bradyrhizobium japonicum*[13] accumulated more PHB than their isogenic nitrogen-fixing parental strains. There is a conflict between rhizobia and legumes over the rate of PHB accumulation, due to the metabolic tradeoff between nitrogen fixation and PHB accumulation. Therefore, PHB biosynthesis and accumulation in species of rhizobia may be controlled to balance the tradeoff, but the mechanism underlying this control has not yet been fully explained.

One of the best studied microorganisms with respect to PHB biosynthesis and accumulation is the Gram-negative bacterium *Ralstonia eutropha*[14]. It synthesizes PHB using the three PHB synthetic genes: *phbA*, which encodes 3-ketoacyl-CoA thiolase; *phbB*, which encodes acetoacetyl CoA reductase; and *phbC*, which encodes the enzyme PHB synthase. PHB degradation, however, is performed by PHB depolymerase, which is encoded by *phaZ*. Phasins, encoded by *phaP*, are a class of low-molecular-mass amphipathic proteins that form a layer at the surface of the PHB granule and stabilize it [15]. The *R. eutropha* possesses at least four *phaP* paralogs identified so far [16]. Expression of the major phasin, encoded by *phaP1*, is regulated by the transcriptional repressor PhaR [17,18]. Under conditions less favorable for PHB biosynthesis, PhaR binds to the *phaP1* promoter region to repress transcription of this gene. After the onset of PHB biosynthesis, when the nascent PHB granules gradually form, PhaR leaves the promoter and binds to the granules so that *phaP1* is transcribed and translated. During the later stages of PHB accumulation, PhaR is estimated to bind no longer to the granules as it is displaced by PhaP1 phasin. The displaced PhaR returns to bind to the *phaP1* promoter and represses transcription again [16].

Most members of the Rhizobiaceae are known to possess single copies of the PHB biosynthesis genes. For instance, strains of *Sinorhizobium meliloti*, the symbionts of alfalfa, regarded as one of the model organisms to study symbiotic nitrogen fixation, are characterized to have a single set of the genes for PHB metabolism, namely *phbA*, *phbB*, *phbC*, and *phaZ*[19,20], whereas two paralogous genes, *phaP1* and *phaP2*, encode functional phasins [21]. On the other hand, strains of *B. japonicum*, the symbionts of soybean, are known to accumulate a large amount of PHB [22], and the *B. japonicum* USDA110 genome was found to contain five paralogs of *phbC*, as well as two paralogs of *phbAB*[23]. This genetic redundancy may suggest a functional importance that has not yet been fully elucidated. In this study, we examined the expression profiles of the paralogs relevant to PHB metabolism in free-living *B. japonicum* cells under PHB accumulating and non-accumulating conditions. Then, we identified the phasin-encoding paralogs from the genomic information [24], and used a variety of tools to investigate their involvement in PHB accumulation.

## Results and discussion

### *B. japonicum* candidate genes involved in metabolism and PHB accumulation

**Figure 1:**
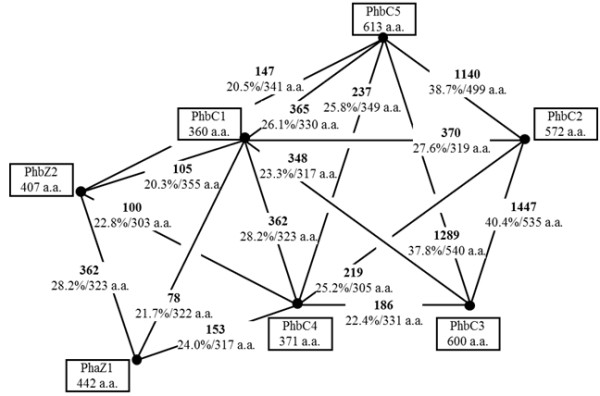
**Similarities among the gene products putatively involved in PHB polymerization/depolymerization.** Similarities were calculated using the FASTA program [26] for each of the indicated pairs. FASTA optimized scores (boldface) and sequence identity (percentage/overlapping amino acid residues) are shown. Size, given as the number of amino acid residues, is indicated beneath the product name.

The genome of *B. japonicum* USDA110 possesses five paralogs of *phbC*, namely *phbC1* (open reading frame blr2885), *phbC2* (blr3732), *phbC3* (bll4360), *phbC4* (bll4548), and *phbC5* (bll6073), as well as two paralogs of *phbA* and *phbB*, *phbA1* (blr3724), *phbA2* (bll0226), *phbB1* (bll3725), and *phbB2* (bll0225) [23]. We predicted that two putative *phaZ* genes from the *B. japonicum* genome [24] encode PHB depolymerases based on their similarities to the *phaZ* of *S. meliloti* (SMc02770 in *S. meliloti* 1021 genome) [25], which had previously been functionally characterized [20]. The results of amino acid sequence comparisons among the products of the *phbC* and *phaZ* paralogs are summarized in Figure 1. The gene products of *phbC2*, *phbC3*, and *phbC5* are remarkably similar to each other. Those of *phbC1* and *phbC4*are shorter but share similarities in the C-terminal regions of *phbC2*, *phbC3*, and *phbC5*. The *phaZ* paralogs were found to share weak similarities to *phbC1*, *phbC4*, and *phbC5*, which may have implications in the enzyme evolution for the reversal reactions of PHB polymerization and depolymerization.

**Figure 2:**
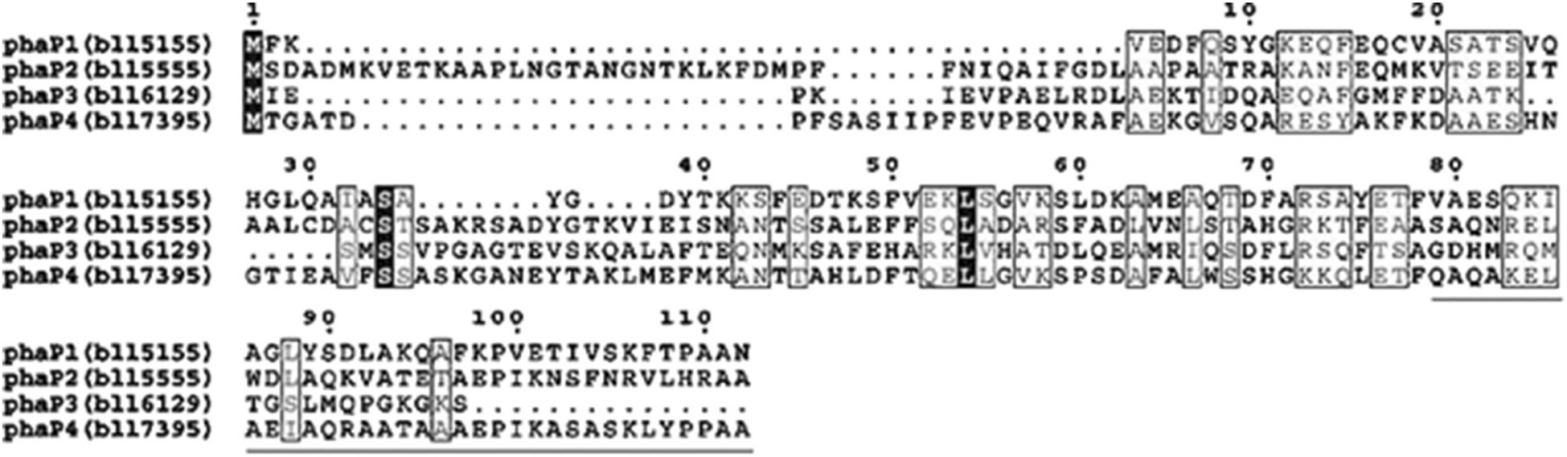
**Alignment of amino acid sequences of predicted PhaP phasins.** White letters on a black background and boxed letters designate conserved and equivalent residues, respectively. The alanine-rich sequence in the C-terminus is underlined beneath the sequence of PhaP4.

Within the *B. japonicum* USDA110 genome, there are seven genes predicted to encode phasins because their deduced amino acid sequences could contain the Phasin_2 motif (http://pfam.sanger.ac.uk/family/PF09361) [27]. Judging from conservation of the motif, we selected four out of the seven genes for this study: *phaP1* (bll5155), *phaP2* (bll5555), *phaP3* (bll6129), and *phaP4* (bll7395). The motifs predicted in the other three putative phasin paralogs were assessed as less reliable (data not shown). These four paralogs are small proteins of 112–161 amino acid residues; an alignment of their amino acid sequences is shown in Figure 2. The putative *phaR* (blr0227), encoding the transcriptional repressor of *phaP*, was deduced by its similarity to the previously identified *phaR* of *S. meliloti*[21].

### Expression profile of the candidate genes in free-living cells

**Figure 3:**
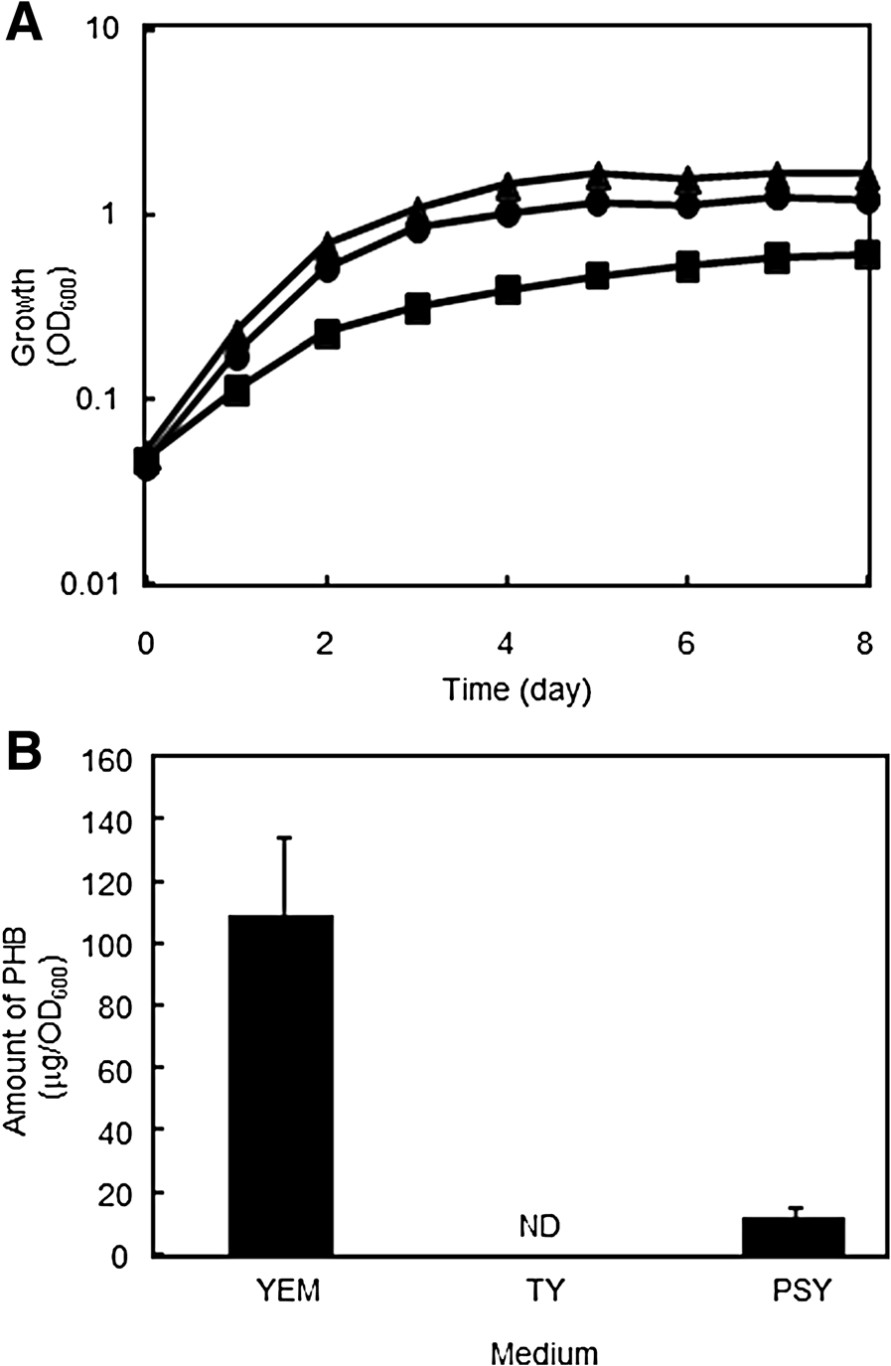
**Growth and PHB accumulation of *B. japonicum *USDA110. (A)** Growth curves for *B. japonicum* USDA110 cells grown in YEM (solid squares), TY (solid circles), and PSY (solid triangles) media. **(B)** Amounts of PHB accumulated. Values are means of three independent results ± SD. ND: not detected.

Cells of *B. japonicum* USDA110 were inoculated into three different media [TY (tryptone yeast), PSY (peptone salts yeast), and YEM (yeast extract mannitol)] and allowed to grow for eight days (Figure 3A). Cells exhibited the slowest growth in YEM, which is rich in carbon sources but poor in nitrogen sources. When the optical density reached 0.8 at 600 nm, cells were harvested, and their intracellular PHB content was measured (Figure 3B). No PHB was detected in the cells grown in TY medium, whereas only a trace of PHB was detected in cells grown in PSY. On the other hand, a substantial amount of PHB was detected in the cells grown in YEM. Replacing mannitol, the carbon source in YEM, with an equivalent concentration of other sugars, including arabinose, mannose, glucose, and sorbitol, resulted in similar levels of PHB accumulation (data not shown). These results suggest that the PHB accumulation does not specifically depend on mannitol, but on the richness of the carbon sources together with a relative lack of nitrogen sources available in the medium. Under nutritional conditions in which carbon sources are in excess relative to nitrogen sources, the intracellular pool of substrates for PHB synthesis, including acetyl CoA and acetate, would be enlarged by less efficient nitrogen assimilation, which may be one of the signals triggering PHB accumulation.

**Figure 4:**
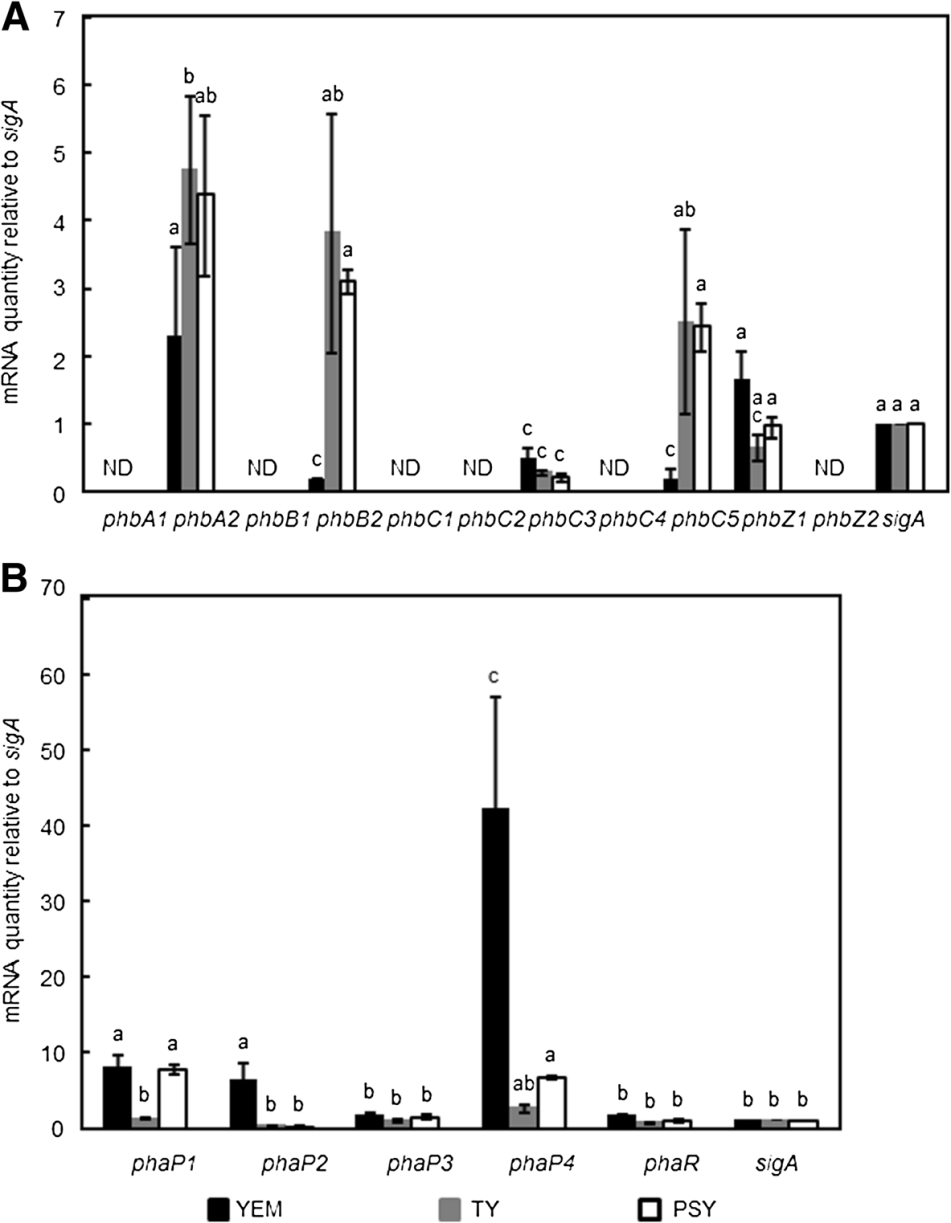
**Transcription profile of the genes deduced to be involved in PHB metabolism and accumulation. (A)** Expression of the genes for PHB biosynthesis and degradation. qRT-PCR analysis was performed as described in the Methods, and data were normalized to constitutively expressed *sigA* as an internal control. Values are means of three independent results ± SD, and those followed by the same letters are not significantly different at the 95% confidence level. ND: no specific PCR product was detected as in the negative control experiment without reverse transcription, and thus was not taken into account for statistic analysis. **(B)** Expression of the four PhaP phasins. qRT-PCR analysis was performed and the results are presented as described for **(A)**.

PHB began to appear in cells cultured in YEM at an optical density of 0.6 at 600 nm (data not shown). We prepared total RNA samples from cells grown in each of the three media, and then subjected the samples to quantitative reverse transcriptase PCR (qRT-PCR) analysis to measure the expression levels of the genes possibly involved in PHB biosynthesis and degradation. Among the genes predicted to be involved in PHB metabolism, we detected transcription of *phbA2*, *phbB2*, *phbC3*, *phbC5*, and *phaZ1*, whereas expression of the others was negligible (Figure 4A), indicating that only one or two of the respective paralogs functioned. Moreover, the levels of transcription of the PHB biosynthetic genes were higher under PHB non-accumulating conditions in TY medium than accumulating conditions in YEM, and thus obviously they were not induced upon PHB accumulation. It was also paradoxical that *phaZ1*, which could be involved in PHB degradation, seemed to be induced under PHB-accumulating conditions.

The transcription profile of *phaP* and *phaR* involved in PHB accumulation was also examined using qRT-PCR (Figure 4B). In contrast to the PHB-metabolic genes, induction of some of the *phaP* encoding putative phasins correlated with PHB accumulation. Among the four *phaP*, *phaP4* was most prominently induced under PHB-accumulating conditions in YEM medium reaching levels up to 40 times greater than that of the control, *sigA*, which encodes the house-keeping sigma factor. These results imply that *phaP4* may play an important role in PHB accumulation. When cultured in YEM, *phaP1* and *phaP2* were induced to levels up to 10 times greater than the control, implying that *phaP1* and *phaP2* may also have roles in PHB accumulation. In PSY medium, both *phaP1* and *phaP4* were induced to lower levels, which may be relevant to the lower PHB accumulation seen in this medium (Figure 3B). On the other hand, expression of *phaR* was kept at a low level and only barely enhanced upon PHB accumulation, which is consistent with the self-regulation model proposed in *R. eutropha*[16]. Transcription of *phaP3* was almost constant and as low as that of *phaR*, and thus this paralog might be irrelevant to PHB accumulation under these conditions.

When all these results are considered, it is conceivable that PHB accumulation in *B. japonicum* during free-living growth may not depend on either the redundancy or expression levels of the genes for PHB synthesis and degradation. Instead, it seems probable that the major mechanism allowing *B. japonicum* to accumulate large amounts of PHB may be the formation of PHB granules stabilized by phasins.

### The four PhaP phasins and PhaR bound to PHB with different affinities

*phaP1*, *phaP2*, *phaP3*, *phaP4*, and *phaR* were cloned individually into *Escherichia coli* and expressed as N-terminally His_6_-tagged fusion proteins. For unknown reason, the His_6_-tag fusions could not be purified by the conventional affinity chromatography. Therefore, the crude extracts of *E. coli* cells containing the fusions were used directly in the PHB binding experiment. Because the N-terminus of each fusion protein contained the same single His_6_-tag, we assumed that each His_6_-tag equally reacts with the anti-His_6_-tag antibody, presumably regardless of fusion partner, and the signal intensities on immunoblots probed for the His_6_-tag were used to represent the amounts of the phasin fusions contained in the extracts.

**Figure 5:**
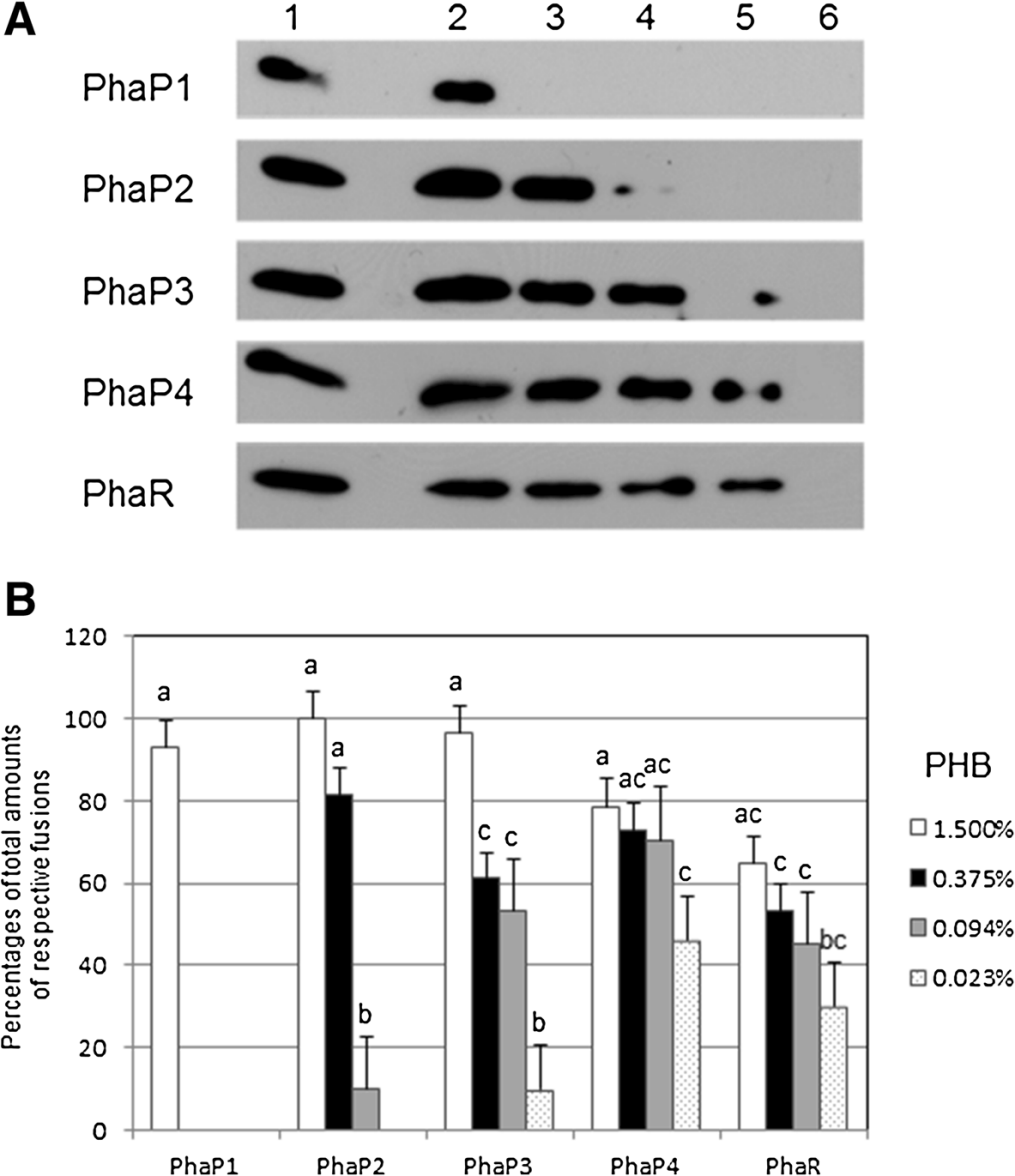
**PHB binding of His**_**6**_**-tag PhaP phasins and His**_**6**_**-tag PhaR *in vitro*. (A)** Immunoblots to detect proteins contained in PHB/protein complexes. The amounts of target protein in the crude extracts were compared to controls, and then fixed to contain the same concentration of each of the His_6_-tag fusions of four PhaP phasins and PhaR. Target proteins were mixed with serially diluted suspensions of PHB, as a fine powder, in test tubes and incubated to allow formation of PHB/protein complexes. The PHB/protein complexes were spun down, washed to remove non-specific proteins, and then subjected to 18% SDS-PAGE followed by the immunoblot analysis as described in the Methods. Total crude extract in a tube (lane 1) and proteins contained in the PHB/protein complexes formed without (lane 6) and with 1.500% (w/v) (lane 2), 0.375% (lane 3), 0.094% (lane 4), and 0.023% (lane 5) PHB are loaded. One set of representative data, from three independent experiments with similar results, is shown. **(B)** Summary of PHB binding assay. Signal intensities on the immunoblots were quantified using ImageJ software [29] and defined as the parameters representing the amounts of the His_6_-tag fusion proteins on the blots. The amounts of His_6_-tag fusions contained in the PHB/protein complexes, formed without (lane 6 in panel **A**) and with 1.500% (w/v) (lane 2), 0.375% (lane 3), 0.094% (lane 4), and 0.023% (lane 5) PHB, are expressed as percentages of total amounts of respective fusions (lane 1). Values are means of three independent results ± SD, and those followed by the same letters are not significantly different at the 95% confidence level.

The crude extracts containing phasin and PhaR fusions, adequately diluted and controlled to give almost the same signal intensities on immunoblots, were mixed with a serially diluted suspension of fine PHB powder in test tubes, and incubated on ice to allow formation of PHB/protein complexes. The insoluble PHB/protein complexes were spun down, washed to remove out non-specific proteins, and then subjected to SDS-PAGE followed by immunoblot analysis. As shown in Figure 5, all four phasin fusions, as well as the PhaR fusion, exhibited some PHB binding. This suggests that their native forms may possess the proposed function of covering the surface of PHB granules in vivo. PhaP4 and PhaR showed the highest affinities to PHB, as they bound it tightly at lower concentrations, whereas the other three had lower affinities. As mentioned above, these four PhaP proteins contain the Phasin_2 motif (http://pfam.sanger.ac.uk/family/PF09361), but only PhaP4 possesses the C-terminal region containing an amino acid sequence stretch very rich in alanine, in which 13 out of 34 residues are alanine (Figure 2). The alanine-rich sequence in the PhaP proteins of *R. eutropha*[28] was proposed to be important for exerting phasin function. This may also be the case with PhaP4 of *B. japonicum*.

**Figure 6:**
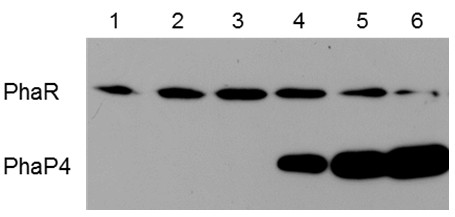
**Competition in PHB binding between His**_**6**_**-tag PhaP4 and His**_**6**_**-tag PhaR.** The amount of crude extract was compared to controls and fixed to contain His_6_-tag PhaR equivalent to 0.094% (w/v) PHB in each of the tubes, and then various amounts of extract containing His_6_-Tag PhaP4 were added and incubated to allow formation of PHB/protein complexes. The complexes were spun down and subjected to the immunoblot analysis described in Figure 5. Lane 1 contains His_6_-tag PhaR alone and no His_6_-tag PhaP4. Concentrations of His_6_-tag PhaR and His_6_-tag PhaP are controlled in the ratios of 4:1 (lane 2), 4:2 (lane 3), 4:4 (lane 4), 4:8 (lane 5), and 4:16 (lane 5). One set of representative data, from three independent experiments with similar results, is shown.

Pötter and colleagues proposed the following mechanism for PHB granule development in *R. eutropha*[16]. When PHB is not produced, PhaR exerts its repressor function by binding DNA and repressing transcription of *phaP1*, which encodes the major phasin. With the onset of PHB accumulation, PhaR leaves the DNA to bind to the neonate PHB droplet, allowing induction of *phaP1*. Then, as more PhaP1 is produced and begins to occupy the surface of the growing PHB granule, PhaR is outcompeted and expelled from the granule and returns to DNA to repress *phaP1* again. In order to determine if this proposed mechanism is also operating in *B. japonicum*, we compared the PHB affinities of PhaP4 and PhaR using an in vitro competition assay. Fixed amounts of PhaR and PHB were mixed in test tubes, and various amounts of PhaP4 were added to the mixture. After incubation, the proteins contained in the insoluble PHB/protein complexes were subjected to the immunoblot analysis described above. As shown in Figure 6, as the amount of PhaP4 increased, more PhaP4 and less PhaR were found in the complexes. These results indicate that PhaP4 and PhaR competed with each other for binding to PHB, and that PhaP4 at higher concentrations could replace PhaR bound to PHB. We have already shown, above, that *phaP4* was most prominently induced upon PHB accumulation (Figure 4B). Taken together, the results obtained in this study suggest that PhaP4 may play the most important role among the four PHB-binding phasins, and could possibly be regulated by PhaR using a mechanism similar to the one proposed in *R. eutropha*.

We have not experimentally assessed the actual repressor function of PhaR; these experiments will be performed and reported later. In addition, to confirm the importance of *phaP4* and *phaR*, we attempted to construct knockout of these, as well as the other *phaP*. However, for unknown reasons, repeated attempts were not successful. We have considered the construction of *B. japonicum* mutants overexpressing these genes to see the effects not only during free-living growth but also during symbiosis with the host plant. The results of these experiments would be reported in the near future.

## Conclusions

*B. japonicum* USDA110 accumulated intracellular PHB during free-living culture in the presence of excess carbon sources together with restricted nitrogen sources. Its genome contains redundant paralogs that could be involved in PHB biosynthesis and degradation, but only one or two of each paralog family was found to be expressed during free-living growth. In addition, expression of the PHB metabolic genes was not correlated with PHB accumulation. Thus, it is conceivable that PHB accumulation during free-living growth is independent of redundancy or expression levels of PHB metabolic genes. Instead, it was found that some of the four *phaP* encoding phasins were induced upon PHB accumulation. All the four phasins exhibited some PHB binding in vitro. PhaP4 showed the highest affinity for PHB and could be responsible for the majority of PhaP function. Furthermore, PhaP4 was able to compete for PHB binding with PhaR, which is its plausible transcriptional repressor and possesses high affinity to PHB. PhaP4 is able to expel PhaR and stabilize the PHB granule. Therefore, in free-living *B. japonicum*, carbon sources in excess relative to nitrogen sources enlarge the pool of substrates for PHB synthesis, such as acetyl-CoA and acetate. This could allow elevation in levels of intracellular PHB, which is recognized by PhaR repressor. This recognition triggers induction of phasins, including PhaP4 and maybe some others. Phasins then autonomously stabilize the accumulated PHB granules. This proposed mechanism resembles the mechanism proposed in *R. eutropha*.

## Methods

### Bacterial strains, plasmids, primers, and culture conditions

Bacterial strains and plasmids used in this study are listed in Table 1. A platinum loop full of glycerol frozen stock culture of *B. japonicum* USDA101 was used to inoculate PSY liquid medium [30] and allowed to grow for five days at 28°C with shaking at 180 rpm. Aliquots of this culture were diluted with YEM [31], TY [19], or PSY media, to an optical density of 0.05 at 600 nm. These three cultures were further incubated at 28°C with shaking at 180 rpm. Strains of *E. coli* were usually maintained at 37°C on LB plates with 50 μg/mL kanamycin or ampicillin added, as required.

**Table 1:** Bacterial strains and plasmids

**Strains and plasmids**	**Relevant genotypes and derivation**	**Source and reference**
*B. japonicum*		
USDA110		24
*E. coli*		
DH5a	*supE44*, *DlacU169*, *hsdR17*, *recA1*, *endA1*, *gyrA96*, *thi-1*, *relA1*	Laboratory stocks
BL21 (DE3)	*F*^*-*^*ompT hsdS*_b_ (*r*_b_^*-*^*m*_b_^*-*^) *gal dcm* (DE3)	Laboratory stocks
Plasmids		
pET-28b	Protein expression vector, kanamycin resistant	Takara Bio
pETPhaP1	pET28b carrying *phaP1*	This work
pETPhaP2	pET28b carrying *phaP2*	This work
pETPhaP3	pET28b carrying *phaP3*	This work
pETPhaR	pET28b carrying *phaR*	This work
pColdII	Protein expression vector, ampicillin resistant	Takara Bio
pColdPhaP4	pColdII carrying *phaP4*	This work

### Quantification of PHB

USDA101 cells in the cultures were harvested by centrifugation, washed once in 50 mM Tris–HCl (pH 8.0) containing 1 M NaCl, and then suspended in 10 mM Tris–HCl (pH 8.0) containing 5 mM 2-mercaptoethanol, 5 mM ethylenediaminetetraacetic acid, 10% (w/v) glycerol, and 0.02 mM phenylmethylsulfonyl fluoride. The cells were subsequently disrupted by sonication in an ice bath. An aliquot (0.1 mL) of the solution was mixed with 1.2 mL of 5% (w/v) sodium hypochlorite, and incubated at 37°C for 1 h. After centrifugation, the pellet was successively washed with 1 mL aliquots of water, acetone, and 99.5% ethanol. PHB contained in the dried pellet was extracted three times with 0.1 mL of chloroform at 50°C, and the chloroform extracts were combined in a tube (0.3 mL in total). After evaporating the chloroform, the remaining substances were dissolved in 1 mL of concentrated H_2_SO_4_, and the absorbance of the solution was measured at 235 nm. Various amounts of commercially available PHB solid powder (Sigma Aldrich, St. Louis, MO) were treated using the procedure described above to produce a standard curve, which was then used to quantify PHB according to the absorbance.

### Plasmid construction and production of the recombinant proteins

DNA fragments corresponding to the coding regions of *phaP1*, *phaP2*, *phaP3*, *phaP4*, and *phaR*, flanked by specific restriction sites, were amplified using PCR from chromosomal DNA of USDA101 using the primer pairs described in Table 2. The DNA fragments were trimmed with the appropriate restriction enzymes and ligated to similarly restricted plasmid vector pET-28b or pColdII, so that each of the coding regions was fused with a His_6_-tag at its N-terminus. After confirming the correct cloning using DNA sequencing, each of the recombinant plasmids was introduced into *E. coli* BL21(DE3), which was allowed to grow in LB liquid medium at 37°C. In the middle of logarithmic growth, the temperature was shifted to 15°C; 30 min later, isopropyl β-D-1-thiogalactopyranoside was added to the culture, which was further incubated for 15 h. The cells in the culture were harvested by centrifugation, washed once in 50 mM potassium phosphate buffer (pH 8.0), 20% (w/v) glycerol, and 0.5 M NaCl, and then suspended in the same solution to be disrupted by sonication in an ice bath. After centrifugation, the supernatant was used in further experiments as the crude extract containing the recombinant protein.

**Table 2:** Oligonucleotide primers

**Oligonucleotide primers**	**Sequence (designed restriction sites are underlined)**
PhaP1 NdeI	5′-*GGAATTCCATATG*ATGTTCAAGGTTGAAGACTT-3′
PhaP1 XhoI	5′-*CCGCTCGAG*TTAGTTGGCGGCCGGGGTGA-3′
PhaP2 NdeI	5′-*GGAATTCCATATG*GTGAGTGATGCCGATATGAA-3′
PhaP2 XhoI	5′-*CCGCTCGAG*TCAAGCAGCCCTATGCAGAA-3′
PhaP3 NdeI	5′-*GGAATTCCATATG*ATGATCGAACCGAAAATCGA-3′
PhaP3 XhoI	5′-*CCGCTCGAG*TCAGGATTTGCCCTTGCCCG-3′
PhaP4 NdeI	5′-*GGAATTCCATATG*ATGACAGGTGCGACTGATCC-3′
PhaP4 HindIII	5′-*CCCAAGCTT*TCAGGCGGCGGGCGGGTAGA-3′
PhaR NdeI	5′-*GGAATTCCATATG*GCGAAATCAGACCAACCCAC-3′
PhaR HindIII	5′-*CCCAAGCTT*CTACTCTTCCTTCTTCGACA-3′
RTphbA1-F	5′-CATCGCCGTCAACAAGGA-3′
RTphbA1-R	5′-CCGCTTCTGCATCTCGAAC-3′
RTphbA2-F	5′-AAGAAGGCCGGCTGGAA-3′
RTphbA2-R	5′-CCATTGACGTTGACCTTGGA-3′
RTphbB1-F	5′-TCGAACTACGACGCCTGTG-3′
RTphbB1-R	5′-ATGCCGTCCTTGGTGATG-3′
RTphbB2-F	5′-CCGAAGGCGTGAAGAAGGT-3′
RTphbB2-R	5′-GAACAGCGAGCCGAGATTG-3′
RTphbC1-F	5′-GCTCTGGGAAAACATCTGGAAC-3′
RTphbC1-R	5′-TTGGTGATGGTGCGGAAA-3′
RTphbC2-F	5′-GGACGACTACGTTGAGGATGG-3′
RTphbC2-R	5′-AATGGCGAGTGCGGATG-3′
RTphbC3-F	5′-ATGACCGCGTCGAACCA-3′
RTphbC3-R	5′-GGCACCTTGACCTTGGAGA-3′
RTphbC4-F	5′-GGCGAAGACAGGCAAACA-3′
RTphbC4-R	5′-CTCCATCCATCCGAACCA-3′
RTphbC5-F	5′-CCGCAAAATTCCCTGGTC-3′
RTphbC5-R	5′-CATCCCTGTCCTTCGCATC-3′
RTphaZ1-F	5′-CCGAAGCAACGCACACA-3′
RTphaZ1-R	5′-ATCCTCGGCACGATTTCC-3′
RTphaZ2-F	5′-GGCACATCAAGCAGCACA-3′
RTphaZ2-R	5′-AGATCCATCACCGCGAAA-3′
RTphaP1-F	5′-ACGGCGACTACACCAAGAAG-3′
RTphaP1-R	5′-GAAGGTCTCGTAGGCGGAAC-3′
RTphaP2-F	5′-TCGCTTTTACCGAGCAGAAC-3′
RTphaP2-R	5′-GTGAACTGGCTACGCAGGA-3′
RTphaP3-F	5′-ATTACGGCACCAAGGTCATC-3′
RTphaP3-R	5′-GTGGAGAGGTTCACGAGGTC-3′
RTphaP4-F	5′-GTGCGACTGATCCATTCTCC-3′
RTphaP4-R	5′-GTCCTTGAACTTGGCGTAGC-3′
RTphaR-F	5′-CTTCGAGCAGGAGAACAAGG-3′
RTphaR-R	5′-GATATTTCGGCACCACCATC-3′
RTsigA-F	5′-CAGGCGAAGGACAAGGAAAA-3′
RTsigA-R	5′-CGTCGGACAGATCGAGCAA-3′

### RNA techniques

Total RNA was extracted from USDA110 cells using TRIZOL RNA isolation reagents (Life Technologies, Carlsbad, CA), treated with DNase I (Roche diagnostics, Basel, Switzerland), and then purified using the RNeasy Mini Kit (Qiagen, Venlo, Netherlands). The qRT-PCR was performed as follows. An aliquot (1 μg) of the total RNA sample was reverse transcribed using the ReverTra Ace qPCR RT Kit (Toyobo, Tokyo, Japan). The resulting cDNA (75 ng) was mixed with THUNDERBIRD SYBR qPCR Mix (Toyobo) and specific primer pairs (RTgene name-F/R listed in Table 2, e.g., RTphbA1-F and RTphbA1-R), and then analyzed using a Thermal Cycler Dice Real Time System MRQ (Takara Bio, Otsu, Japan) according to the suppliers’ procedures.

### PHB binding assay and immunoblot analysis

The crude extract containing the recombinant protein was adequately diluted and mixed with a suspension containing various amounts of crystalline PHB solid powder (Sigma Aldrich) in 10 mM Tris–HCl (pH 7.5). The mixture was incubated on ice for 90 min, and then centrifuged to pellet the PHB/protein complexes. After washing with 10 mM Tris–HCl (pH 7.5), the pellet was suspended in loading buffer and applied to an 18% SDS-PAGE gel. Proteins separated by electrophoresis were transferred onto a polyvinylidene fluoride membrane that was subsequently probed with an Anti-His6 (2) antibody (Roche) and goat anti-mouse IgM-HRP (Santa Cruz Biotech, Santa Cruz, CA), and visualized with ImmunoStar LD (Wako Pure Chemical Industries, Osaka, Japan).

### Statistical analysis

Microsoft Excel Spreadsheet has been used for data processing. When needed, data were subjected to one-way analysis of variance followed by the Tukey–Kramer multiple comparison test.

## Abbreviations

PHB: Poly-β-hydroxybutyrate; PSY: Peptone salts yeast; qRT-PCR: Quantitative reverse-transcriptase PCR; SDS-PAGE: SDS-polyacrylamide gel electrophoresis.

## Competing interests

The authors declare that they have no competing interests.

## Authors’ contributions

Conception and design of the study: KY. Acquisition of data: YT and TS. Analysis and interpretation of data: KT. Drafting the article: KY. Revising it critically for important intellectual content: KT and ST. Final approval of the version to be submitted: All the co-authors. All authors read and approved the final manuscript.
